# Work-Life Balance of Collaborative Statisticians and Methodologists in Multidisciplinary Settings

**DOI:** 10.1001/jamanetworkopen.2026.7479

**Published:** 2026-04-27

**Authors:** Tolulope T. Sajobi, Olayinka I. Arimoro, Ayooluwanimi P. Okikiolu, Oluwagbohunmi A. Awosoga, Meng Wang, Lawrence Mbuagbaw, Lehana Thabane

**Affiliations:** 1 Department of Community Health Sciences & O’Brien Institute for Public Health, University of Calgary, Calgary, Alberta, Canada; 2 Department of Clinical Neurosciences, University of Calgary, Calgary, Alberta, Canada; 3 Faculty of Health Sciences, University of Lethbridge, Lethbridge, Alberta, Canada; 4 Department of Health Research Methods, Evidence, and Impact, McMaster University, Hamilton, Ontario, Canada; 5 Research Institute of St Joe’s Hamilton, St Joseph’s Healthcare Hamilton, Hamilton, Ontario, Canada; 6 Faculty of Health Sciences, University of Johannesburg, Johannesburg, South Africa

## Abstract

**Importance:**

Collaborative statisticians and methodologists are essential members of multidisciplinary teams, yet little is known about how their diverse responsibilities, including technical analysis, project leadership, and domain-specific consultation across multiple concurrent projects, affect their work-life balance and professional well-being.

**Objective:**

To assess perceptions of work-life balance and identify individual, job-related, and organizational factors associated with work-life balance among collaborative statisticians and methodologists working in multidisciplinary settings.

**Design, Setting, and Participants:**

This cross-sectional survey study collected data from November 2022 to May 2024, with analysis conducted in December 2024. Anonymous electronic surveys were distributed through international professional statistical societies, including organizations in North America, Europe, Africa, South America, and Asia. Participants were recruited via a convenience sample through society email listservs and newsletters. Eligible respondents were statisticians, data scientists, or methodologists working in multidisciplinary collaborative settings who had been continuously employed full-time during the previous year.

**Main Outcomes and Measures:**

Work-life balance was assessed using the 15-item Work-Life Balance Self-Assessment Scale, which consists of 3 subscales: Work Interference With Personal Life, Personal Life Interference With Work, and Work/Personal Life Enhancement. Regression analyses were used to identify the demographic, organizational, and discipline-specific factors associated with respondents’ perception of work-life balance.

**Results:**

Of 627 individuals who accessed the survey, 450 (71.8%) met eligibility criteria and completed the survey. Of the 450 respondents who completed the survey, the mean (SD) age was x (y) years, 240 (53.7%) were female, and 270 (60.0%) worked in academia; 143 (31.8%) reported dissatisfaction with their work-life balance, and an additional 47 (10.4%) reported neutral satisfaction. Factors associated with higher work interference scores included unmanageable workload (mean difference, 0.70; 95% CI, 0.48 to 0.92), work-related health issues (mean difference, 0.58; 95% CI, 0.33 to 0.83), inability to complete work during regular hours (mean difference, 0.56; 95% CI, 0.34 to 0.78), and lack of organizational support for work-life balance (mean difference, 0.48; 95% CI, 0.24 to 0.73). Organizational flexibility to attend to personal matters (mean difference, −0.33; 95% CI, −0.64 to −0.01) and adequate compensation (mean difference, −0.29; 95% CI, −0.49 to −0.09) were associated with lower interference scores.

**Conclusions and Relevance:**

These findings suggest that targeted educational initiatives, effective mentorship, and wellness-focused organizational policies can foster sustainable work-life balance among statisticians and methodologists in multidisciplinary settings.

## Key Points

**Question:**

Which factors are associated with work-life balance among collaborative statisticians and methodologists in multidisciplinary settings?

**Findings:**

In this cross-sectional survey of 450 professionals from multiple countries, 31.8% reported dissatisfaction with their work-life balance, and an additional 10.4% reported neutral satisfaction. Years of professional experience, work-related health disability, organizational support, compensation adequacy, workload manageability, and flexibility to attend to personal matters were significantly associated with work-life balance subscale scores.

**Meaning:**

These findings suggest that organizational policies supporting workplace flexibility, manageable workloads, and professional development may help improve work-life balance among statisticians and methodologists.

## Teaser

This cross-sectional study looks at the roles of collaborative statisticians and methodologists on multidisciplinary teams to investigate associations between their diverse responsibilities and their work-life balance.

## Introduction

Collaborative statisticians and methodologists are indispensable professionals in multidisciplinary settings across many industries, including academia, government, life sciences, and businesses. Within these multidisciplinary settings, their roles and job responsibilities are usually related to study design and data analysis. In recent years, these roles have expanded beyond the traditional technical responsibilities to encompass several aspects and phases of research projects, requiring a well-balanced blend of technical, administrative, and domain-relevant skills.^1,2,3^ For example, the multifaceted responsibilities of collaborative academic statisticians often include teaching, grant writing, mentoring, supervising graduate students, conducting statistical research, and supporting clinical and translational research, in addition to maintaining their independent research programs in statistical science research. Similarly, those embedded within for-profit industries and government might focus heavily on study design, preparing data for stringent regulatory submissions, regulatory review of submitted claims, conducting rigorous data analysis, providing expert consulting to internal and external stakeholders, and overseeing complex project management. Their activities are meticulously aligned with the strategic business objectives of their respective organizations.

Regardless of the work setting, collaborative statisticians and methodologists are typically minority groups within organizations, as they are usually embedded in interdisciplinary teams working alongside other domain experts.^4^ Given that they shoulder a broad range of job responsibilities in multidisciplinary teams, they are usually tasked with supporting multiple projects, with each one having separate expectations and deadlines. When not properly managed, this increasing workload can result in substantial burnout and emotional fatigue, ultimately compromising their work-life balance.^5,6,7^

Although much has been written and published on work-life balance in other disciplines,^8,9^ there is a dearth of research on the work-life balance of collaborative statisticians and methodologists in multidisciplinary settings. This study aims to address this gap by examining the extent to which these professionals experience work-life balance challenges in their roles and identifying personal and organizational factors associated with work-life balance outcomes. Understanding challenges faced by these professionals is not merely an academic exercise; it has practical implications for retaining talent, fostering innovation, and ensuring the continued integrity of scientific research and decision-making in multidisciplinary settings. We hypothesized that organizational factors, such as workload manageability and institutional support, would be more associated with work-life balance than individual demographic characteristics.

## Methods

### Study Design, Setting, and Participants

This international cross-sectional study involved the collection of primary data through anonymous electronic surveys. Eligible respondents included adult statisticians, data scientists, or methodologists who work in multidisciplinary and/or collaborative settings and are currently or have been continuously employed (full-time) over the past year. For the purposes of this study, a multidisciplinary setting was defined as a work environment in which statisticians or methodologists collaborate with professionals from other disciplines (eg, subject matter experts) on research or analytical projects. This includes, but is not limited to, statisticians embedded within academic research teams, clinical trial units, clinical departments, research institutes, pharmaceutical or biotech companies, government agencies, and contract research organizations. The study was reviewed and approved by the University of Calgary Conjoint Health Research Ethics Board. Electronic informed consent was obtained from all participants prior to survey completion. This study followed the Strengthening the Reporting of Observational Studies in Epidemiology (STROBE) reporting guidelines for cross-sectional studies.

### Data Sources, Measures, and Outcomes

Eligible participants were invited to complete a cross-sectional electronic survey that consists of several questions that include details about their sociodemographic characteristics (eg, age, sex and/or gender, years of professional practice, industry of practice, race, region of practice, and mode of work), work responsibilities as collaborative statisticians in multidisciplinary settings, recognition of their contributions on work projects, understanding of work-life balance, self-assessment of work-life balance, personal strategies for managing work-life balance, and satisfaction with work life balance, satisfaction with compensation, and work-related health disability.

#### Work-Life Balance Self-Assessment Survey

The Work-Life Balance Self-Assessment Survey is a 15-item measure of work-life balance proposed by Hayman,^10^ who adapted it from an originally developed 19-item measure developed to capture employee work-life balance.^11^ The survey consists of 15 items, each scored on a 7-point Likert scale, ranging from 1 (not at all) to 7 (all the time). The 15 items can be aggregated into 3 domains: Work Interference With Personal Life (WIPL), Personal Life Interfering With Work (PLIW), and Work/Personal Life Enhancement (WPLE) subscales. The WIPL subscale assesses how work impacts personal life (score range, 1.0-6.6, with higher scores indicating greater interference or worse work-life balance). The PLIW subscale examines how personal commitments impact work (score range, 1.0-6.2, with higher scores indicating greater interference or worse work-life balance). The WPLE subscale assesses how work and personal life positively influence each other (score range, 1.0-7.0, with higher scores indicating greater enhancement or better work-life balance). Previous studies have confirmed its validity and reliability in other populations.^12,13^

#### Satisfaction With Work-Life Balance

This is a single-item 7-point Likert-like self-reported measure of satisfaction with work-life balance. Participants were asked to respond to the following question: “Taking into account all aspects of your work and life in the past 12 months, please indicate your response to this statement: I am satisfied with my work-life balance. (1) strongly disagree, (2) disagree, (3) somewhat disagree, (4) neutral, (5) somewhat agree, (6) agree, (7) strongly agree.”

The initial draft survey was piloted with 5 collaborative statisticians to assess content and face validity. The revised survey was then distributed electronically through several international professional statistical and data science societies between November 2022 and May 2024, including the Statistical Society of Canada, the American Statistical Association, the International Society for Clinical Biostatistics, the Statistical Society of Australia, the Society for Clinical Trials, the International Chinese Statistical Association, and many other national statistical associations in Africa, South America, and Asia. The survey link was distributed via email listservs and newsletters of participating organizations and administered electronically using Qualtrics^14^ as the data collection platform.

### Statistical Analysis

Of the 627 individuals who clicked on the survey link, only respondents who self-identified as working in multidisciplinary settings and who completed the survey are included in these analyses (eFigure in Supplement 1). Descriptive statistics, including mean (SD), median (IQR), frequencies, and percentages, were used to summarize the sociodemographic, individual, job-related, and industry and/or organization characteristics of study participants as appropriate. Race and ethnicity were self-reported by participants using categories defined by the investigators based on commonly used classifications in health research. These categories included Asian, Black or African or Afro-Caribbean, Hispanic or Latino or Latina, Indigenous peoples, White, Other (with option to specify), and prefer not to answer. Race and ethnicity were categorized as White, any other racial or ethnic group, and prefer not to answer to describe the study sample, but were not included in the analyses of the factors impacting work-life balance perceptions. Missing data were assessed for all variables. When there is a low proportion of missing data (<5% for all variables), complete case analysis was used for the regression models without imputation.

Unadjusted mean subscale scores by participant characteristics and organizational support factors were reported. Multiple linear regression was used to identify factors associated with each work-life balance subscale score. The estimated regression coefficients (β̂) represent the adjusted difference in mean subscale scores between comparison groups, holding all other covariates constant. For categorical variables, coefficients indicate the difference in mean score relative to the reference category. Positive coefficients indicate higher subscale scores, which for the WIPL and PLIW subscales reflect greater interference (ie, worse work-life balance), while for the WPLE subscale, positive coefficients reflect greater enhancement (ie, better work-life balance). To explore potential differences between work settings, unadjusted mean subscale scores were reported cross-tabulated with years of experience and stratified regression analyses were conducted separately for respondents in academic and nonacademic settings. A sensitivity analysis was conducted, excluding respondents who reported that their work had caused health or disability issues, to assess the robustness of findings in the population without work-related health disability. All regression analyses were performed at 5% level of significance. All analyses were conducted in R software version 4.4.1 (R Project for Statistical Computing).^15^

## Results

Of the 450 respondents included in this analysis, 240 (53.7%) were female, and 232 (51.9%) were at least 45 years of age. Four hundred ten respondents (91.7%) held advanced graduate degrees (eg, MSc or PhD), and 256 (57.3%) were practicing in North America. Additionally, 270 (60.0%) were practicing in academic settings (Table). More than one-half of the respondents had more than a decade of experience as collaborative statisticians and methodologists, while 277 (61.8%) worked in a hybrid mode.

**Table.** Characteristics of Study Respondents

Characteristic	Respondents, No. (%) (N = 450)
Sex^a^	
Female	240 (53.7)
Male	198 (44.3)
Others	2 (0.4)
Prefer not to answer^b^	7 (1.6)
Age group, y^a^	
18-24	8 (1.8)
25-34	75 (16.8)
35-44	121 (27.1)
45-54	114 (25.5)
≥55	118 (26.4)
Prefer not to answer^b^	11 (2.5)
Education^a^	
Undergraduate and/or others	37 (8.3)
Graduate	410 (91.7)
Race^a^	
White	308 (68.9)
Any race other than White	116 (26.0)
Prefer not to answer^b^	23 (5.1)
Profession	
Statistician	349 (77.6)
Data scientist	62 (13.8)
Methodologist	23 (5.1)
Others	16 (3.6)
Duration of professional experience, y	
<5	60 (13.3)
5-10	73 (16.2)
11-20	132 (29.3)
>20	180 (40.0)
Prefer not to answer^b^	5 (1.1)
Mode of practice^a^	
Fully remote	96 (21.4)
In-person	75 (16.7)
Hybrid	277 (61.8)
Region of practice^a^	
Europe	123 (27.5)
North America	256 (57.3)
Others	68 (15.2)
Industry of practice	
Academic	270 (60.0)
Nonacademic	173 (38.4)
Prefer not to answer^b^	7 (1.6)
Health disability due to work-related duties during the last 12 mo	76 (16.9)
Organizational support for work-life balance^a^	257 (57.1)
Able to complete most of my work during official work hours	206 (45.8)
Flexibility to attend to urgent family/personal life matters without fear of reprisals^a^	395 (87.8)
Access to organization’s wellness resources^a^	287 (63.8)
Recognition of contributions in multidisciplinary settings	387 (86.0)
Manageable and realistic work demands and responsibilities	270 (60.0)
Able to speak up about unrealistic work expectations without fear of reprisals^a^	292 (64.9)
Recognition and appreciation of workload by direct supervisor and others on the team	293 (65.1)
Well compensated^a^	277 (61.6)
I am satisfied with my work-life balance	
Somewhat disagree, disagree, or strongly disagree	143 (31.8)
Neutral	47 (10.4)
Somewhat agree, agree. or strongly agree	260 (57.8)
Work Life Balance Self-Assessment Survey Subscale scores, median (IQR)^c^	
WIPL	3.7 (2.9-4.4)
PLIW	2.5 (1.8-3.2)
WPLE	4.0 (3.7-5.0)

Abbreviations: PLIW, Personal Life Interference with Work; WIPL, Work Interference with Personal Life; WPLE, Work Personal Life Enhancement.

^a^ Data were missing for sex (3 respondents [(0.7%]), age (3 respondents [0.7%]), education (3 respondents [0.7%]), race and ethnicity (3 respondents [0.7%]), mode of practice (2 respondents [0.4%]), and region (3 respondents [0.7%]). Missing values range between 1 (0.2%) and 6 (1.3%) for organizational support items.

^b^ Respondents selecting prefer not to say were excluded from regression analyses for the relevant variable.

^c^ The WIPL subscale scores range from 1.0 to 6.6; PLIW subscale scores range from 1.0 to 6.2; and WPLE subscale scores range from 1.0 to 7.0. For WIPL and PLIW, higher scores indicate greater interference (worse work-life balance). For WPLE, higher scores indicate greater enhancement (better work-life balance).

Seventy-six (16.8%) respondents reported having work-related health disability, 257 (57.1%) reported organizational support, 395 (87.8%) reported flexibility to attend to urgent personal and/or family situations without fear of any reprisals, and 387 (86.0%) reported being recognized for their work contributions. However, only 287 (63.8%) respondents had access to work-life wellness resources with their organization, 270 (60.0%) reported having manageable and realistic work demands and expectations, 293 (65.1%) reported recognition and appreciation of workload by their direct supervisor and others on the team, and 206 (45.8%) reported being able to complete their work responsibilities during the official work hours. Overall, only 260 (57.8%) respondents considered their work-life balance to be optimal, 143 (31.8%) reported dissatisfaction with their work-life balance, and 47 (10.4%) reported neutral satisfaction (Table). Missing data were minimal (0.4%-0.7% for demographics; 0.2%-1.3% for organizational support items).

Participants’ understanding of work-life balance, valued role aspects, strategies employed, and perceived supportive approaches are presented in eTable 1 in Supplement 1. The most commonly employed strategies for achieving work-life balance were priority setting, time management, and delegation. When asked about approaches that would support healthy work-life balance, respondents most frequently endorsed work flexibility, manageable workload, and support from immediate supervisors.

For WIPL and PLIW subscales, lower scores indicate better work-life balance. For the WPLE subscale, higher scores indicate better work-life balance. Unadjusted mean subscale scores stratified by demographic characteristics and organizational support factors are presented in eTable 2 in Supplement 1. Notably, respondents with manageable workloads had substantially lower WIPL scores (3.08 vs 4.28) and higher WPLE scores (4.53 vs 3.65) than those with unmanageable workloads.

**Figure 1.**
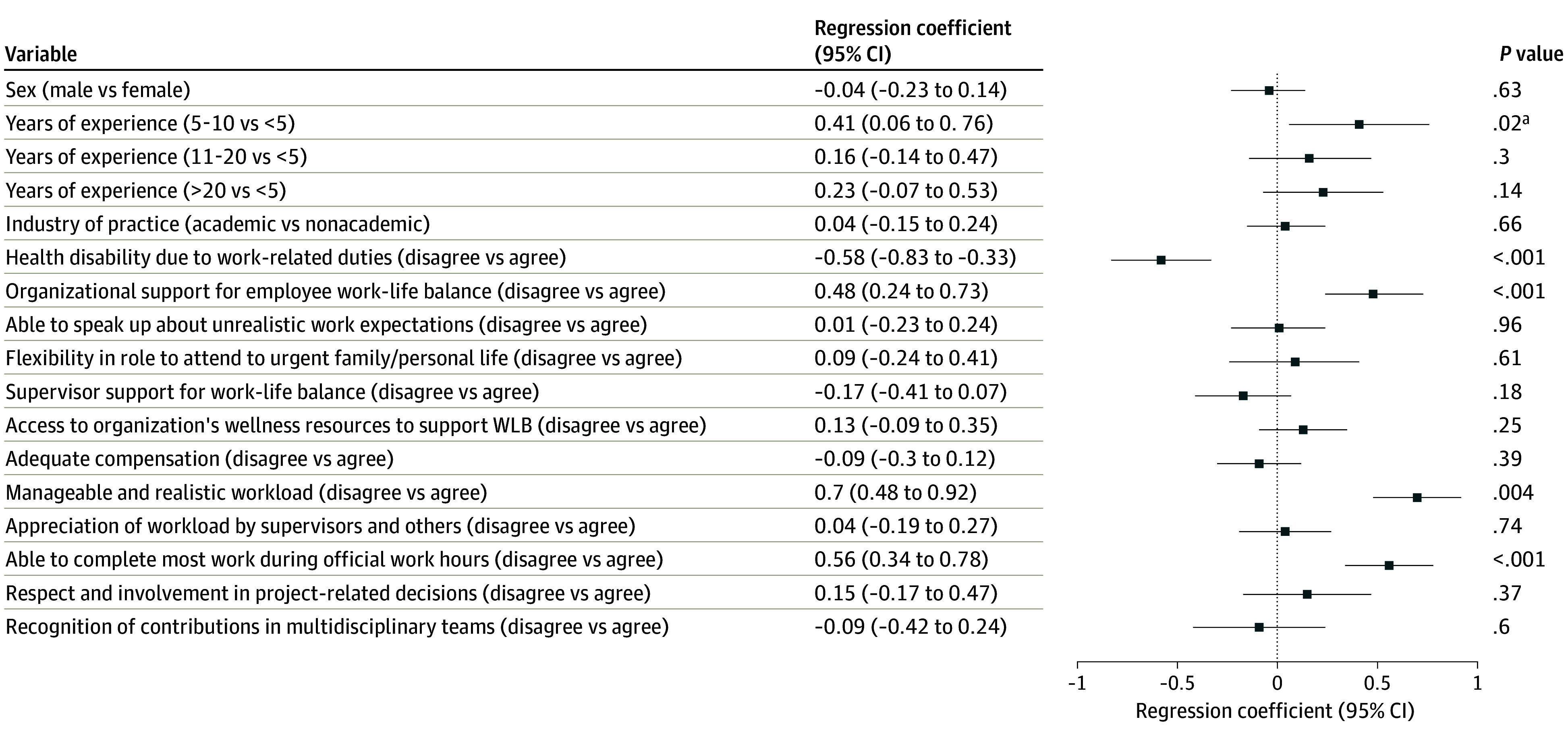
Dot Plot of Adjusted Regression Coeffecients (95% CI) for Factors Associated With Work Interference With Personal Life Subscale Data are mean differences in subscale scores. Abbreviation: WLB indicates work life balance. ^a^Denotes statistical significance.

**Figure 2.**
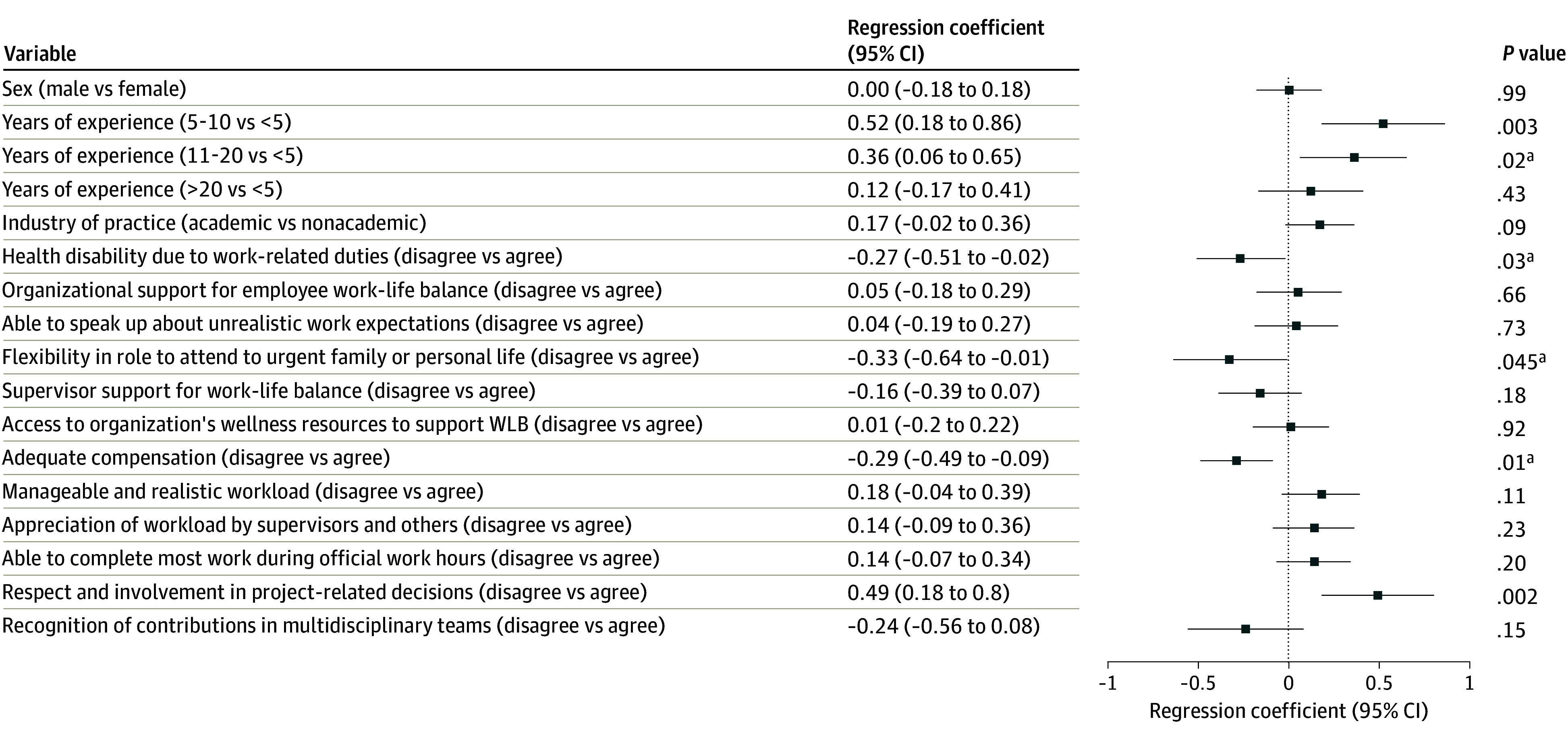
Dot Plot of Adjusted Regression Coefficients (95% CI) for Factors Associated With Personal Life Interference With Work Subscale Data are mean differences in subscale scores. Abbreviation: WLB indicates work life balance. ^a^Denotes statistical significance.

**Figure 3.**
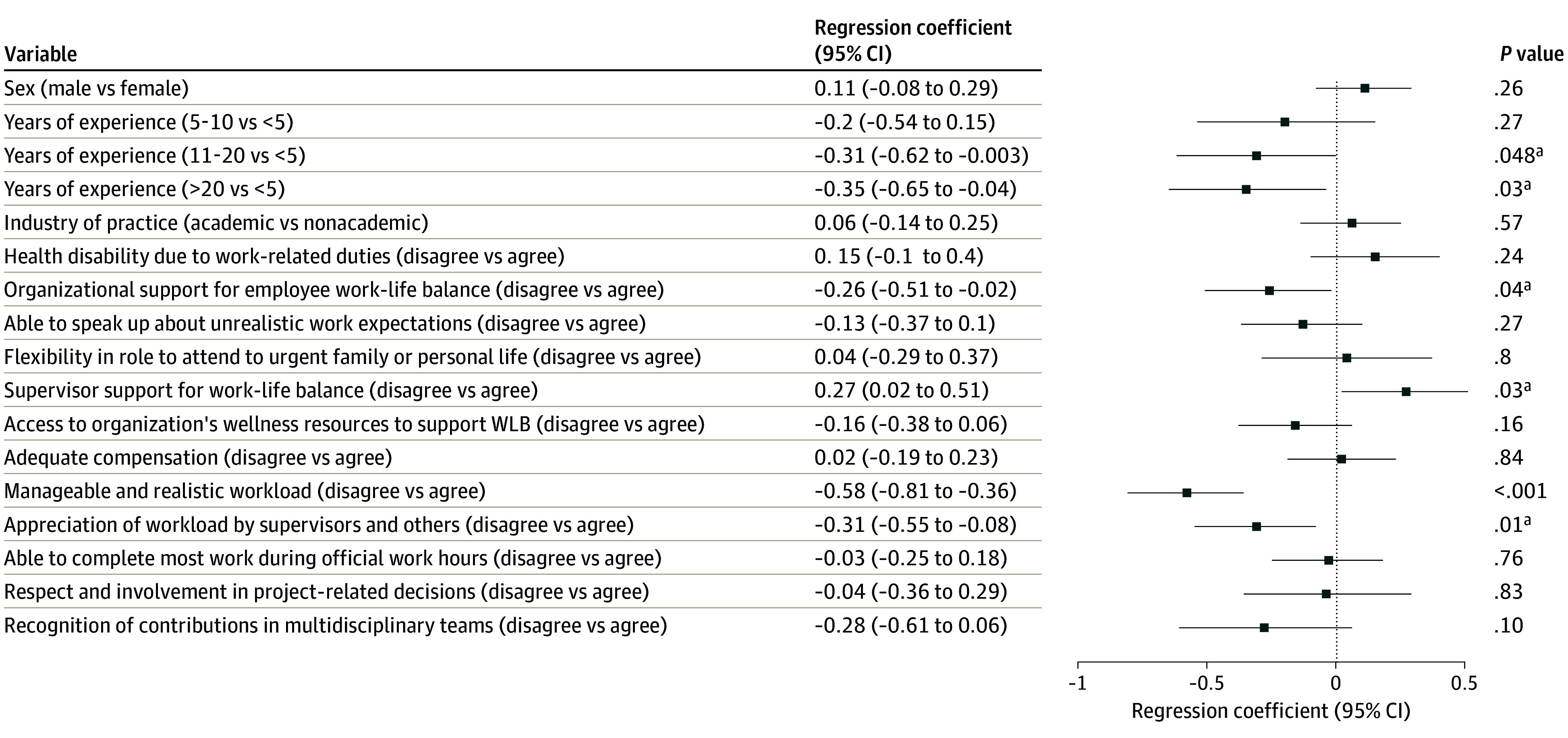
Dot Plot of Adjusted Regression Coefficients (95% CI) for Factors Associated With Work and/or Personal Life Enhancement Subscale Data are mean differences in subscale scores. Abbreviation: WLB indicates work life balance. ^a^Denotes statistical significance.

The adjusted regression results are presented in Figures 1, 2, and 3. For the WIPL subscale (Figure 1), respondents with 5 to 10 years of experience reported significantly higher scores than those with less than 5 years (mean difference, 0.41; 95% CI, 0.06 to 0.76; *P* = .02). Work-related health disability (mean difference, 0.58; 95% CI, 0.33 to 0.83; *P* < .001), lack of organizational support for work-life balance (mean difference, 0.48; 95% CI, 0.24 to 0.73; *P* < .001), unmanageable workload (mean difference, 0.70; 95% CI, 0.48 to 0.92; *P* < .001), and inability to complete work during regular hours (mean difference, 0.56; 95% CI, 0.34 to 0.78; *P* < .001) were also associated with higher interference scores. For the PLIW subscale (Figure 2), respondents with 5 to 10 years (mean difference, 0.52; 95% CI, 0.18 to 0.86; *P* = .002) and 11 to 20 years of experience (mean difference, 0.36; 95% CI, 0.06 to 0.65; *P* = .02) reported higher scores. Work-related health disability (mean difference, 0.27; 95% CI, 0.02 to 0.51; *P* = .03) and lack of respect in project decisions (mean difference, 0.49; 95% CI, 0.18 to 0.80; *P* = .002) were associated with higher interference, while organizational flexibility for personal matters (mean difference, −0.33; 95% CI, −0.64 to −0.01; *P* = .04) and adequate compensation (mean difference, −0.29; 95% CI, −0.49 to −0.09; *P* = .01) were associated with lower interference. For the WPLE subscale (Figure 3), respondents with 11 to 20 years (mean difference, −0.31; 95% CI, −0.62 to −0.003; *P* = .04) and greater than 20 years of experience (mean difference, −0.35; 95% CI, −0.65 to −0.04; *P* = .03) reported lower enhancement scores. Lack of organizational support (mean difference, −0.26; 95% CI, −0.51 to −0.02; *P* = .04), unmanageable workload (mean difference, −0.58; 95% CI, −0.81 to −0.36; *P* < .001), and lack of supervisor and/or others appreciation for workload (mean difference, −0.31; 95% CI, −0.55 to −0.08; *P* = .01) were associated with lower enhancement scores.

Stratified regression analyses by work setting revealed that the association between years of experience and work-life balance differed between academic and nonacademic respondents (eTables 3-4 in Supplement 1). Among academic statisticians, midcareer professionals (5-20 years) reported significantly higher WIPL scores than early-career colleagues, whereas among nonacademic statisticians, experience was primarily associated with declining WPLE scores among senior professionals (>20 years). Organizational support factors, particularly workload manageability and ability to complete work during regular hours, were consistently associated with better outcomes across both settings (eTable 4 in Supplement 1). In sensitivity analyses excluding the 76 respondents (16.9%) who reported work-related health or disability issues, the pattern of associations remained consistent with the primary analysis (eTable 5 in Supplement 1). Organizational support factors, including workload manageability (mean difference in WIPL score, 0.70; 95% CI, 0.46-0.94), organizational encouragement of work-life balance (mean difference in WIPL score, 0.48; 95% CI, 0.22-0.75), and ability to complete work during regular hours (mean difference in WIPL score, 0.59; 95% CI, 0.36-0.82), remained significantly associated with WIPL scores.

## Discussion

In a cross-sectional study of statisticians and methodologists working in multidisciplinary settings, the majority of respondents (57.8%) reported satisfaction with their work-life balance. The high proportion of respondents with long careers, access to hybrid work arrangements, and flexibility for personal matters suggests that many statisticians work in relatively supportive environments. Nevertheless, nearly one-third reported dissatisfaction, indicating room for improvement. Respondents’ perception of their work-life balance was associated with the stage of their professional career, presence of work-related health disability, organizational support for work-life balance, adequate compensation, workload, and flexibility to attend to urgent personal and/or family matters without fear of reprisals. To our knowledge, this is the first study to investigate the work-life balance of collaborative statisticians and methodologists working in multidisciplinary settings.

These findings are consistent with the existing body of evidence from the literature on the importance of individual^16,17,18^ and organizational factors that shape perceptions of work-life balance.^19,20,21,22,23,24^ The prevalence of reported dissatisfaction with their work-life balance in this study is comparable with those observed in other professions.^25,26,27,28^ Longitudinal studies of US physicians have documented work-life balance satisfaction declining from 48.5% in 2011 to 40.9% in 2014, with physicians having nearly twice the risk of burnout and work-life dissatisfaction compared with workers in other fields after adjusting for work hours and education level.^25,26^ Among university faculty, studies have reported burnout prevalence ranging from 35% to 37%, with workload and institutional support showing significant associations with work-life balance outcomes.^27,28^ The factors we identified as associated with work-life balance among statisticians, including workload manageability, organizational support, and workplace flexibility, are consistent with those reported in the physician and academic faculty literature.^25,26,27,28^

These findings hold several implications for statistical and data science practice in multidisciplinary settings. Given the observed associations between organizational factors and work-life balance, educational workshops and coaching focused on workload management, boundary setting, and negotiation skills may be worth exploring as potential strategies to support statisticians and methodologists.^29,30^ Such initiatives could be offered through in-house professional development programs or through statistical and data science professional societies. Prior research in other fields suggests that effective mentoring is positively associated with healthy work-life balance, career satisfaction, and improved overall wellness.^30,31,32,33,34^ Mentorship programs that pair senior professionals with early-career and midcareer colleagues may provide opportunities for sharing insights on navigating high-pressure work situations, understanding organizational norms, and setting realistic boundaries between work and personal life.^35^ The nonlinear relationship between years of experience and work-life balance outcomes may reflect the competing influences of career stage. Midcareer professionals may face peak demands from simultaneous pressures of establishing research programs, mentoring, seeking promotion, and family responsibilities, while more senior professionals may have greater autonomy but also increased administrative and leadership burdens.^36,37,38^ The setting-specific patterns we observed suggest these dynamics operate differently across work environments.

### Limitations

This study has some notable limitations. An integral aspect of work-life balance is the associated outcome of personal life on work and vice versa. However, this survey did not collect data on respondents’ personal lives (eg, marital status, family composition, and caregiving responsibilities) or detailed work environment characteristics (eg, team size or organizational type), which are known to influence work-life balance. Furthermore, while these analyses did not reveal any significant gender differences in perceptions of work-life balance, the extent to which female respondents accounted for their unpaid invisible labor (eg, caring for children, domestic work, and caregiving for aged parents) and how it impacts their work-life balance was also not examined. In addition, more than 65% of study respondents had a decade or more of experience in their careers and are likely to be established midlevel professionals or senior leaders in their organizations. It is not clear to what extent the perception of work-life balance presented in this study represents the broader population of all statisticians and methodologists who work in multidisciplinary settings.

Although our findings suggest that organizational factors may play an important role in work-life balance perceptions, our cross-sectional design precludes causal inference. Future longitudinal research and intervention studies are needed to determine whether modifications to organizational policies, mentorship programs, or professional development opportunities could improve work-life balance outcomes for this population. Furthermore, data on whether respondents held managerial or supervisory positions were not collected, which limits the ability to examine this potentially important factor. The association between greater years of experience and poorer work-life balance outcomes in some subscales may partially reflect the increased administrative and supervisory responsibilities that often accompany career advancement; future studies should explicitly assess managerial responsibilities and their relationship to work-life balance in this population.

## Conclusions

In this cross-sectional survey study of 450 collaborative statisticians and methodologists working in multidisciplinary settings across multiple countries, while a majority reported satisfaction with their work-life balance, nearly one-third reported dissatisfaction. Work-related factors, such as an unmanageable workload, inadequate organizational support, and a lack of support from supervisors, were associated with poor work-life balance. These findings may inform future research examining interventions to support work-life balance in this population.

## Notes

Supplement 1. eFigure. Flowchart of respondents’ initiation and completion of survey eTable 1. Descriptive analysis of the questions on work-life balance and job role by satisfaction eTable 2. Unadjusted mean work-life balance subscale scores by participant characteristics and organizational support factors eTable 3. Unadjusted mean work-life balance subscale scores by work setting and years of experience eTable 4. Adjusted regression coefficients for work-life balance subscales stratified by work settings eTable 5. Sensitivity analysis: adjusted regression coefficients excluding respondents with work-related health issues (n=374)

Supplement 2. Data Sharing Statement
