# Supplementary material for: Work-Life Balance of Collaborative Statisticians and Methodologists in Multidisciplinary Settings
Source: JAMA Netw Open. 2026 Apr 27;9(4):e267479. doi: 10.1001/jamanetworkopen.2026.7479 (PMC13122407; doi:10.1001/jamanetworkopen.2026.7479)
Supplement: Supplement 1. — eFigure. Flowchart of respondents’ initiation and completion of survey eTable 1. Descriptive analysis of the questions on work-life balance and job role by satisfaction eTable 2. Unadjusted mean work-life balance subscale scores by participant characteristics and organizational support factors eTable 3. Unadjusted mean work-life balance subscale scores by work setting and years of experience eTable 4. Adjusted regression coefficients for work-life balance subscales stratified by work settings eTable 5. Sensitivity analysis: adjusted regression coefficients excluding respondents with work-related health issues (n=374) [file jamanetwopen-e267479-s001.pdf]

Supplemental Online Content

Sajobi TT, Arimoro OI, Okikiolu AP, et al. Work-life balance of collaborative statisticians and methodologists in multidisciplinary settings. *JAMA Netw Open.* 2026;9(4):e267479. doi:10.1001/jamanetworkopen.2026.7479

eFigure. Flowchart of respondents' initiation and completion of survey

eTable 1. Descriptive analysis of the questions on work-life balance and job role by satisfaction

eTable 2. Unadjusted mean work-life balance subscale scores by participant characteristics and organizational support factors

eTable 3. Unadjusted mean work-life balance subscale scores by work setting and years of experience

eTable 4. Adjusted regression coefficients for work-life balance subscales stratified by work setting

eTable 5. Sensitivity analysis: adjusted regression coefficients excluding respondents with work-related health issues (n=374)

This supplemental material has been provided by the authors to give readers additional information about their work.

© 2026 Sajobi TT et al. *JAMA Network Open*.

eFigure. Flowchart of respondents' initiation and completion of survey

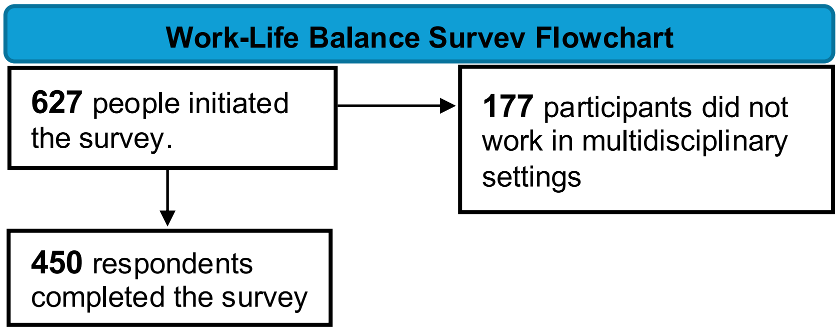

© 2026 Sajobi TT et al. *JAMA Network Open*.

eTable 1. Descriptive analysis of the questions on work-life balance and job role by satisfaction

	n (%)[a]	
	Not Satisfied	Satisfied
**What is your understanding of work-life balance in the past 12 months?**		
Equal contributions	44 (23.2)	79 (30.4)
Ability to prevent work from negatively impacting other aspects of life and vice versa	151 (79.5)	196 (75.4)
Adequate management of stress at work	101 (53.2)	146 (56.2)
*A dynamic construct that varies depending on several factors**	107 (56.3)	144 (55.4)
Priority setting	76 (40.0)	119 (45.8)
Time management	88 (46.3)	148 (56.9)
Others	6 (3.2)	17 (6.5)
**What do you like the most about your role in the past 12 months?**		
Flexible working hours (Flexibility)	121 (63.7)	191 (73.5)
Salary and/or benefits (Compensation)	85 (44.7)	133 (51.2)
Opportunity to contribute in a meaningful way (Meaningful contribution)	143 (75.3)	219 (84.2)
Opportunity to collaborate with others (Collaboration)	129 (67.9)	204 (78.5)
Opportunity to learn new things (Learning)	131 (68.9)	199 (76.5)
Leadership opportunity (Leadership)	57 (30.0)	94 (36.2)
Control over my work, such as project scope and time allocation (Independence)	49 (25.8)	107 (41.2)
Others	12 (6.3)	14 (5.4)
**Which of these strategies have you employed to achieve work-life balance in the past 12 months?**		
Priority setting	155 (81.6)	228 (87.7)
Time management	130 (68.4)	201 (77.3)
Project management skills	71 (37.4)	131 (50.4)
Saying NO to more work	118 (62.1)	136 (52.3)
Education	25 (13.2)	56 (21.5)
Delegation	97 (51.1)	127 (48.8)
Mentorship	46 (24.2)	84 (32.3)
Negotiating for flexible work arrangement (Negotiating flexibility)	32 (16.8)	65 (25.0)
Job resignation	17 (8.9)	12 (4.6)
Transfer to other units/roles in the organization (Work transfer)	12 (6.3)	16 (6.2)
Seeing a therapist/coach (Therapy)	37 (19.5)	35 (13.5)
Others	6 (3.2)	10 (3.8)
**Which of these approaches is helpful for supporting a healthy work-life balance?**		
Access to organizational wellness resources	49 (25.8)	77 (29.6)
Clearly defined boundaries for work and non-work times	115 (60.5)	183 (70.4)
Compensation for extra hours worked	76 (40.0)	80 (30.8)
Educational programs on wellness and work-life balance (e.g., workshops, webinars, coaching)	35 (18.4)	78 (30.0)
Manageable workload	151 (79.5)	204 (78.5)

© 2026 Sajobi TT et al. *JAMA Network Open*.

Organizational support	114 (60.0)	163 (62.7)
Support from immediate supervisor	131 (68.9)	187 (71.9)
Work flexibility (e.g., remote work, flex days, etc.)	150 (78.9)	235 (90.4)
Others	10 (5.3)	15 (5.8)
**Which of these describe your responsibilities in your current role in the past 12 months?**		
Consulting	117 (61.9)	157 (60.6)
Computer simulations/Computing	82 (43.4)	99 (38.2)
Data Management/Data engineering/Data processing	88 (46.6)	109 (42.1)
Data analysis/modelling	162 (85.7)	218 (84.2)
Grant applications	113 (59.8)	119 (45.9)
Literature review	90 (47.6)	116 (44.8)
Manuscript/Report preparation	150 (79.4)	197 (76.1)
Meeting attendance	155 (82.0)	190 (73.4)
Predictive analytics	39 (20.6)	68 (26.3)
Project management	100 (52.9)	106 (40.9)
Protocol writing	91 (48.1)	117 (45.2)
Statistical programming	128 (67.7)	166 (64.1)
Study design	140 (74.1)	176 (68.0)
Supervision	127 (67.2)	133 (51.4)
Travel	86 (45.5)	96 (37.1)
Training/Teaching	122 (64.6)	156 (60.2)
Team/Organizational leadership	110 (58.2)	118 (45.6)
Others	11 (5.8)	15 (5.8

Note: *Several factors include time of the day, marital status, family composition, stage of career, etc). The stem question is: *"Taking into account all aspects of your work and life in the past 12 months"* [a]The n (%) does not add up to 100%, but it shows how many respondents endorsed that option.

© 2026 Sajobi TT et al. *JAMA Network Open*.

eTable 2. Unadjusted mean work-life balance subscale scores by participant characteristics and organizational support factors

Variable	Category	n	WIPL, Mean (SD)	PLIW, Mean (SD)	WPLE, Mean (SD)
Sex	Female	240	3.63 (1.16)	2.54 (0.92)	4.09 (1.06)
	Male	198	3.48 (1.21)	2.48 (1.01)	4.28 (1.07)
Years of experience	<5 years	60	3.05 (1.11)	2.13 (0.86)	4.60 (0.99)
	5-10 years	73	3.76 (1.23)	2.76 (1.08)	4.19 (1.03)
	11-20 years	132	3.60 (1.21)	2.64 (0.97)	4.07 (1.09)
	>20 years	180	3.60 (1.14)	2.44 (0.87)	4.09 (1.06)
Region of practice	North America	256	3.47 (1.19)	2.53 (0.91)	4.13 (1.05)
	Europe	123	3.78 (1.09)	2.63 (0.94)	4.01 (1.06)
	Other regions	68	3.46 (1.30)	2.22 (1.12)	4.62 (1.05)
Industry of practice	Non-academic	173	3.28 (1.24)	2.36 (0.96)	4.29 (1.15)
	Academic	270	3.74 (1.12)	2.62 (0.94)	4.1 (1.01)
Health disability due to work-related duties	Disagree	374	3.40 (1.15)	2.45 (0.92)	4.26 (1.06)
	Agree	76	4.34 (1.05)	2.80 (1.06)	3.79 (1.00)
Organizational support for employee work-life balance	Disagree	191	4.07 (1.12)	2.57 (0.94)	3.83 (1.00)
	Agree	257	3.17 (1.08)	2.47 (0.97)	4.43 (1.04)
Able to speak up about unrealistic work expectations	Disagree	157	3.98 (1.13)	2.57 (0.91)	3.84 (1.04)
	Agree	292	3.33 (1.16)	2.48 (0.98)	4.36 (1.04)
Flexibility in role to attend to urgent family/personal life	Disagree	53	4.05 (1.29)	2.33 (0.94)	3.90 (1.20)
	Agree	395	3.49 (1.16)	2.53 (0.96)	4.22 (1.04)
Supervisor support for work-life balance	Disagree	135	3.87 (1.13)	2.45 (0.89)	4.03 (1.08)
	Agree	309	3.43 (1.19)	2.54 (0.98)	4.23 (1.05)
Access to organization's wellness resources to support work-life balance	Disagree	160	3.94 (1.20)	2.54 (1.00)	3.89 (1.00)
	Agree	287	3.35 (1.12)	2.50 (0.93)	4.33 (1.07)
Adequate compensation	Disagree	172	3.81 (1.19)	2.45 (0.92)	3.93 (1.05)
	Agree	277	3.40 (1.15)	2.55 (0.98)	4.33 (1.05)
Manageable and realistic workload	Disagree	180	4.28 (0.88)	2.73 (0.87)	3.65 (0.96)
	Agree	270	3.08 (1.12)	2.37 (0.99)	4.53 (0.99)
Appreciation of workload by supervisors and others	Disagree	157	4.02 (1.07)	2.67 (0.91)	3.76 (0.98)
	Agree	293	3.31 (1.17)	2.43 (0.97)	4.40 (1.04)
Able to complete most work during official work hours	Disagree	244	4.03 (1.08)	2.65 (0.93)	3.93 (1.04)
	Agree	206	2.99 (1.05)	2.35 (0.97)	4.47 (1.02)
Respect and Involvement in project-related decisions	Disagree	75	3.95 (1.04)	2.81 (0.95)	3.75 (1.00)
	Agree	375	3.48 (1.20)	2.45 (0.95)	4.26 (1.06)
Recognition of contributions in multidisciplinary teams	Disagree	63	3.98 (1.08)	2.66 (1.00)	3.60 (1.03)
	Agree	387	3.49 (1.19)	2.49 (0.95)	4.27 (1.04)

© 2026 Sajobi TT et al. *JAMA Network Open*.

Note: WIPL = Work Interference with Personal Life; PLIW = Personal Life Interference with Work; WPLE = Work Personal Life Enhancement; SD = Standard deviation; WLB = Work-life balance. The stem question for the organizational support questions is: *"Based on your experience in the past 12 months, please indicate your response to the following statement."* For WIPL and PLIW, higher scores indicate greater interference (worse work-life balance). For WPLE, higher scores indicate greater enhancement (better work-life balance).

eTable 3. Unadjusted mean work-life balance subscale scores by work setting and years of experience

Work Setting	Years of Experience	n	WIPL Mean	PLIW Mean	WPLE Mean
**Academic**	<5 years	29	2.88	2.13	4.54
	5-10 years	46	4.12	2.90	3.98
	11-20 years	80	3.94	2.79	3.95
	>20 years	113	3.67	2.49	4.15
**Non-academic**	<5 years	30	3.22	2.13	4.67
	5-10 years	25	3.17	2.58	4.55
	11-20 years	52	3.09	2.42	4.26
	>20 years	65	3.50	2.32	4.01

Note: WIPL = Work Interference with Personal Life; PLIW = Personal Life Interference with Work; WPLE = Work Personal Life Enhancement.

© 2026 Sajobi TT et al. *JAMA Network Open*.

eTable 4. Adjusted regression coefficients for work-life balance subscales stratified by work setting

Variable	Academic Setting			Non-academic Setting		
	WIPL (95% CI)	PLIW (95% CI)	WPLE (95% CI)	WIPL (95% CI)	PLIW (95% CI)	WPLE (95% CI)
Sex (Male vs Female)	-0.11 (-0.33, 0.11)	0.15 (-0.07, 0.38)	0.05 (-0.17, 0.27)	0.07 (-0.26, 0.40)	-0.30 (-0.61, 0.00)*	0.13 (-0.20, 0.47)
Years of experience (5-10 vs <5)	0.66 (0.23, 1.09)**	0.56 (0.13, 0.99)*	-0.17 (-0.60, 0.26)	0.10 (-0.51, 0.70)	0.30 (-0.26, 0.86)	-0.20 (-0.83, 0.42)
Years of experience (11-20 vs <5)	0.43 (0.04, 0.82)*	0.45 (0.05, 0.84)*	-0.21 (-0.60, 0.19)	-0.15 (-0.67, 0.36)	0.19 (-0.28, 0.67)	-0.41 (-0.93, 0.12)
Years of experience (>20 vs <5)	0.29 (-0.09, 0.67)	0.14 (-0.24, 0.52)	-0.11 (-0.50, 0.27)	0.31 (-0.21, 0.84)	0.03 (-0.45, 0.52)	-0.77 (-1.30, -0.23)**
Health disability due to work-related duties (Disagree vs Agree)	-0.48 (-0.77, -0.18)**	-0.14 (-0.44, 0.15)	0.31 (0.01, 0.61)*	-0.71 (-1.20, -0.22)**	-0.45 (-0.90, 0.00)	-0.10 (-0.60, 0.40)
Organizational support for employee work-life balance (Disagree vs Agree)	0.42 (0.14, 0.70)**	-0.08 (-0.36, 0.20)	-0.31 (-0.59, -0.03)*	0.53 (0.05, 1.01)*	0.17 (-0.27, 0.62)	-0.37 (-0.86, 0.13)
Able to speak up about unrealistic work expectations (Disagree vs Agree)	0.64 (0.38, 0.89)***	0.25 (0.00, 0.51)*	-0.54 (-0.80, -0.29)***	0.65 (0.20, 1.10)**	-0.06 (-0.47, 0.36)	-0.63 (-1.09, -0.17)**
Flexibility in role to attend to urgent family/personal life (Disagree vs Agree)	0.52 (0.26, 0.79)***	0.27 (0.01, 0.54)*	0.01 (-0.26, 0.27)	0.77 (0.38, 1.17)***	-0.05 (-0.41, 0.31)	0.00 (-0.40, 0.41)
Supervisor support for work-life balance (Disagree vs Agree)	-0.15 (-0.53, 0.23)	-0.49 (-0.87, -0.10)*	0.00 (-0.39, 0.38)	0.48 (-0.16, 1.12)	0.05 (-0.55, 0.64)	0.00 (-0.66, 0.66)
Access to organization's wellness resources to support WLB (Disagree vs Agree)	-0.25 (-0.52, 0.02)	0.00 (-0.27, 0.27)	0.22 (-0.05, 0.49)	0.00 (-0.51, 0.51)	-0.56 (-1.03, -0.09)*	0.43 (-0.10, 0.95)
Adequate compensation (Disagree vs Agree)	0.06 (-0.20, 0.33)	0.04 (-0.22, 0.31)	0.00 (-0.27, 0.27)	0.34 (-0.05, 0.73)	0.08 (-0.28, 0.44)	-0.43 (-0.83, -0.03)*
Manageable and realistic workload (Disagree vs Agree)	-0.02 (-0.27, 0.23)	-0.15 (-0.41, 0.10)	0.07 (-0.18, 0.33)	-0.18 (-0.56, 0.20)	-0.38 (-0.74, -0.03)*	-0.04 (-0.44, 0.35)
Appreciation of workload by supervisors and others (Disagree vs Agree)	0.14 (-0.13, 0.41)	0.07 (-0.20, 0.34)	-0.31 (-0.59, -0.04)*	-0.20 (-0.65, 0.25)	0.37 (-0.05, 0.78)	-0.24 (-0.70, 0.23)
Able to complete most work during official work hours (Disagree vs Agree)	0.14 (-0.24, 0.52)	0.50 (0.12, 0.88)*	-0.08 (-0.46, 0.30)	0.12 (-0.49, 0.73)	0.35 (-0.21, 0.92)	0.00 (-0.63, 0.63)
Respect and Involvement in project-related decisions (Disagree vs Agree)	-0.06 (-0.43, 0.32)	-0.21 (-0.58, 0.17)	-0.37 (-0.75, 0.00)	-0.03 (-0.71, 0.65)	-0.21 (-0.84, 0.42)	-0.08 (-0.78, 0.61)

Note: *p < 0.05; **p < 0.01; ***p < 0.001. WIPL = Work Interference with Personal Life; PLIW = Personal Life Interference with Work; WPLE = Work Personal Life Enhancement; CI = Confidence interval; WLB = Work-life balance. The coefficients represent adjusted differences in mean subscale scores compared to the reference category. For WIPL and PLIW, positive coefficients indicate worse work-life balance; for WPLE, negative coefficients indicate worse work-life balance.

© 2026 Sajobi TT et al. *JAMA Network Open*.

eTable 5. Sensitivity analysis: adjusted regression coefficients excluding respondents with work-related health issues (n=374)

Variable	WIPL (95% CI)	p	PLIW (95% CI)	p	WPLE (95% CI)	p
Sex (Male vs Female)	-0.11 (-0.32, 0.09)	0.27	-0.06 (-0.25, 0.13)	0.55	0.10 (-0.11, 0.31)	0.34
Years of experience (5-10 vs <5)	0.35 (-0.03, 0.72)	0.07	0.38 (0.03, 0.73)	0.03	-0.28 (-0.67, 0.10)	0.14
Years of experience (11-20 vs <5)	0.08 (-0.24, 0.41)	0.61	0.27 (-0.03, 0.57)	0.07	-0.28 (-0.61, 0.05)	0.10
Years of experience (>20 vs <5)	0.21 (-0.11, 0.53)	0.20	0.06 (-0.23, 0.36)	0.67	-0.35 (-0.68, -0.02)	0.04
Industry of practice (Academic vs Non-academic)	0.05 (-0.17, 0.27)	0.64	0.20 (0.00, 0.40)	0.05	0.13 (-0.10, 0.35)	0.26
Organizational support for employee work-life balance (Disagree vs Agree)	0.48 (0.22, 0.75)	<.001	0.07 (-0.18, 0.32)	0.59	-0.29 (-0.56, -0.01)	0.04
Able to speak up about unrealistic work expectations (Disagree vs Agree)	0.03 (-0.23, 0.29)	0.83	0.00 (-0.24, 0.24)	1.00	-0.12 (-0.39, 0.15)	0.38
Flexibility in role to attend to urgent family/personal life (Disagree vs Agree)	-0.01 (-0.40, 0.39)	0.98	-0.34 (-0.71, 0.02)	0.07	0.09 (-0.31, 0.49)	0.66
Supervisor support for work-life balance (Disagree vs Agree)	-0.14 (-0.41, 0.13)	0.30	-0.18 (-0.43, 0.07)	0.15	0.30 (0.03, 0.58)	0.03
Access to organization's wellness resources to support WLB (Disagree vs Agree)	0.13 (-0.12, 0.38)	0.31	0.08 (-0.16, 0.31)	0.52	-0.11 (-0.37, 0.15)	0.41
Adequate compensation (Disagree vs Agree)	-0.02 (-0.26, 0.21)	0.84	-0.34 (-0.56, -0.12)	0.003	0.06 (-0.18, 0.31)	0.61
Manageable and realistic workload (Disagree vs Agree)	0.70 (0.46, 0.94)	<.001	0.19 (-0.03, 0.42)	0.09	-0.62 (-0.87, -0.37)	<.001
Appreciation of workload by supervisors and others (Disagree vs Agree)	0.10 (-0.16, 0.36)	0.45	0.19 (-0.05, 0.44)	0.11	-0.29 (-0.55, -0.02)	0.03
Able to complete most work during official work hours (Disagree vs Agree)	0.59 (0.36, 0.82)	<.001	0.16 (-0.05, 0.38)	0.14	-0.02 (-0.26, 0.22)	0.87
Respect and Involvement in project-related decisions (Disagree vs Agree)	0.20 (-0.15, 0.55)	0.27	0.57 (0.24, 0.89)	<.001	-0.08 (-0.44, 0.28)	0.67
Recognition of contributions in multidisciplinary teams (Disagree vs Agree)	-0.09 (-0.45, 0.28)	0.64	-0.27 (-0.61, 0.07)	0.12	-0.36 (-0.73, 0.02)	0.06

Note: p = p-value; WIPL = Work Interference with Personal Life; PLIW = Personal Life Interference with Work; WPLE = Work Personal Life Enhancement; CI = Confidence interval; WLB = Work-life balance. Analysis excludes 76 respondents (16.9%) who agreed that their work had caused health or disability issues. The regression coefficients represent adjusted differences in mean subscale scores compared to the reference category. For WIPL and PLIW, positive coefficients indicate worse work-life balance; for WPLE, negative coefficients indicate worse work-life balance.

© 2026 Sajobi TT et al. *JAMA Network Open*.
