# Supplementary material for: Work-Life Balance of Collaborative Statisticians and Methodologists in Multidisciplinary Settings
Source: JAMA Netw Open. 2026 Apr 27;9(4):e267479. doi: 10.1001/jamanetworkopen.2026.7479 (PMC13122407; doi:10.1001/jamanetworkopen.2026.7479)
Supplement: Supplement 2. — Data Sharing Statement [file jamanetwopen-e267479-s002.pdf]

Data Sharing Statement

Sajobi. Work-Life Balance of Collaborative Statisticians and Methodologists in Multidisciplinary Settings. *JAMA Netw Open*. Published April 27, 2026.
doi:10.1001/jamanetworkopen.2026.7479

Data
Data available: Yes
Data types: Deidentified participant data, Data dictionary
How to access data: The datasets used and/or analysed during the current study are available from the corresponding author (ttsajobi@ucalgary.ca) on reasonable request and with appropriate permission from the University of Calgary Conjoint Health Research Ethics Board.
When available: beginning date: 06-29-2026

Supporting Documents
Document types: None

Additional Information
Who can access the data: Researchers whose proposed use of the data has been approved
Types of analyses: For any analyses
Mechanisms of data availability: With a signed data access agreement
Any additional restrictions: None
